# Hepatitis E Virus (HEV) Seroprevalence in Cryptogenic Cirrhosis: From Evidence of High Frequency to the Impact on Disease Progression

**DOI:** 10.3390/medicina61112014

**Published:** 2025-11-11

**Authors:** Serkan Yaraş, Osman Özdoğan, Seda Tezcan Ülger, Gönül Aslan, Eyüp Naci Tiftik, Orhan Sezgin

**Affiliations:** 1 Department of Internal Medicine (Gastroenterology), Faculty of Medicine, Mersin University, 33343 Mersin, Türkiye; osmanozdogan2000@gmail.com (O.Ö.); orhansezgin@mersin.edu.tr (O.S.); 2 Department of Medical Microbiology, Mersin University School of Medicine, 33343 Mersin, Türkiye; tezcanseda@mersin.edu.tr (S.T.Ü.); drgaslan@mersin.edu.tr (G.A.); 3 Department of Hematology, Mersin University School of Medicine, 33343 Mersin, Türkiye; ntiftik@mersin.edu.tr

**Keywords:** cryptogenic cirrhosis, hepatitis E virus, prevalence, fibrosis, liver disease, prognosis

## Abstract

*Background and Objectives*: The Hepatitis E Virus (HEV) is increasingly recognized as a cause of chronic infection in immunocompromised patients, but its precise role in cryptogenic cirrhosis (CC) is unclear. CC is defined as liver cirrhosis in which all known causes, including viral, autoimmune, metabolic, and alcohol-related etiologies, have been meticulously excluded. We aimed to address this gap by definitively assessing HEV’s etiological contribution in CC through seroprevalence comparison and evaluating its long-term prognostic impact on disease progression and adverse clinical outcomes. *Materials and Methods*: This is a retrospective, single-center, observational, and longitudinal cohort study, conducted between July 2017 and June 2025. The study included 52 CC patients, whose diagnosis was strictly confirmed by excluding all known etiologies, and 900 healthy blood donors from the same region. CC patients were retrospectively followed for five years to assess long-term clinical outcomes. We compared HEV seropositive and seronegative patients for accelerated disease progression (assessed by follow-up MELD-Na scores) and cirrhosis-related death. We employed multivariable logistic regression to adjust for demographic confounders in the prevalence comparison and multivariable COX regression for survival analysis to determine the independent prognostic role of HEV seropositivity. *Results*: The anti-HEV IgG seroprevalence in CC patients (42.3%) was significantly higher than in healthy donors (12.8%) (*p* < 0.001). Multivariable logistic regression confirmed CC status as an independent predictor of HEV seropositivity (Adjusted OR = 6.142, *p* < 0.001). During the five-year follow-up, the cirrhosis-related death rate was significantly higher in the anti-HEV IgG positive group (36.4% vs. 13.4%; *p* = 0.047), and their follow-up MELD-Na score was significantly higher (*p* = 0.029). However, multivariable COX analysis did not sustain anti-HEV IgG positivity as an independent risk factor for death (*p* = 0.294). *Conclusions*: HEV exposure is independently and significantly higher in CC patients. While anti-HEV IgG positivity correlates with higher mortality and accelerated disease progression in univariable analysis, its lack of independent prognostic significance suggests it may primarily function as a marker for a more advanced stage of CC or underlying immune dysfunction. Further rigorous prospective studies are necessary to precisely define HEV’s long-term prognostic role and evaluate its impact on disease progression.

## 1. Introduction

The Hepatitis E Virus (HEV), a small, single-stranded RNA virus belonging to the Hepeviridae family, represents a significant global public health concern [1]. Historically, HEV was classified as a non-enveloped virus during its fecal–oral transmission route, but it can exist in a “quasi-enveloped” form within the bloodstream, complicating its classification and recognition [2]. HEV infection has been primarily associated with endemic regions across Central and Southeast Asia, as well as North and West Africa, where transmission predominantly occurs via the fecal–oral route [3]. However, in developed and non-endemic countries, the primary transmission route involves zoonosis through the consumption of raw or undercooked meat products and, less commonly, blood transfusions. While HEV typically results in an acute, self-limiting disorder, the clinical course can be severe in specific populations, notably in pregnant women [4]. Crucially, while typically self-limiting, the high variability in HEV’s clinical presentation, ranging from asymptomatic infection to acute liver failure, necessitates vigilance across different patient demographics.

HEV is classified into at least eight distinct genotypes (Genotypes 1–8), with G1 and G2 typically causing waterborne outbreaks in developing countries, while G3 and G4 are zoonotic, primarily transmitted through contaminated meat products in industrialized regions. The clinical course of HEV infection is highly variable; it is crucial to note that the vast majority of immunocompetent patients remain asymptomatic and experience spontaneous viral clearance without the need for specific treatment [5,6].

For decades, HEV infection was exclusively perceived as an acute and transient illness. However, the last two decades have witnessed a paradigm shift, recognizing HEV as a cause of chronic hepatitis in immunocompromised individuals [7,8]. The development of chronic HEV infection—confirmed by persistent viremia for over six months—is strongly associated with genotypes 3 and 4 [5]. Initial reports documented chronic HEV infection in patients receiving immunosuppressive treatment following solid-organ transplantation [7,8]. Subsequent findings confirmed that chronic HEV infection can also occur in HIV-infected patients and in individuals undergoing chemotherapy for hematological malignancies [7,9,10]. In these vulnerable populations, persistent HEV viremia is a serious clinical entity, often leading to chronic hepatocellular damage and rapid progression to fibrosis and cirrhosis [11,12]. The progression from persistent infection to chronic liver inflammation and subsequent rapid advancement to cirrhosis underscores the necessity of screening and early intervention in these at-risk groups [13]. This established link between HEV persistence and accelerated liver pathology provides a strong biological rationale for hypothesizing that HEV exposure may worsen the trajectory of existing cirrhosis.

Recent surveillance and reporting efforts have increasingly hypothesized that unrecognized HEV infection may be the underlying source of chronic hepatitis of unknown etiology in certain patient groups [14,15]. This highlights a critical diagnostic gap: the long-term clinical significance of HEV co-existence with established cirrhotic diseases is not well characterized. Cryptogenic Cirrhosis (CC), defined as cirrhosis after the exclusion of all known etiologies (viral, autoimmune, metabolic, or alcoholic), represents a significant and growing clinical challenge, accounting for a substantial proportion of end-stage liver disease cases. CC is associated with poor cumulative survival and often lacks etiology-specific treatment options, underscoring the urgency of identifying contributing risk factors and prognostic modifiers [16,17]. Given that cirrhosis itself induces a state of immune dysfunction, now termed ‘cirrhosis-associated immune dysfunction’, we hypothesize that this compromised immune status creates a favorable environment for HEV persistence, which, in turn, contributes to the progression of the underlying liver disease [18]. Despite this strong pathophysiological hypothesis, studies comparing the frequency of HEV seropositivity in CC patients with that in a healthy population are scarce, and almost no data exists describing the long-term clinical course or prognostic impact of HEV-positive cases within the CC cohort. Thus, the specific etiological role of HEV in CC and its subsequent long-term impact on patient prognosis remain unclear. In this study, our primary aim was twofold: First, to definitively establish the etiological role of HEV in CC by comparing its seroprevalence with that in a local healthy donor cohort. Second, to investigate the association of HEV seropositivity with long-term prognostic markers and clinical outcomes, specifically focusing on differences in disease progression and major adverse clinical outcomes (such as decompensation and mortality) between HEV-positive and HEV-negative patients during the follow-up period.

## 2. Materials and Methods

### 2.1. Study Design and Population

#### 2.1.1. Cryptogenic Cirrhosis Patients

T This was a single-center, retrospective cohort study conducted at the Gastroenterology Clinic of Mersin University School of Medicine, designed to evaluate the impact of known Hepatitis E Virus (HEV) exposure (anti-HEV IgG positivity) on long-term clinical outcomes in patients already diagnosed with cryptogenic cirrhosis (CC). The study population consisted of consecutive patients who met the defined criteria and had been definitively diagnosed with CC following an exhaustive diagnostic workup prior to June 2020, and for whom anti-HEV IgG serology testing was available on file (i.e., performed at some point for any clinical reason). Patient data were collected retrospectively from hospital charts (medical files and laboratory databases) starting from the date of HEV serology testing and covering a 5-year follow-up period. The study assessed both the baseline clinical and laboratory data (at the time of HEV serology) and clinical outcome data recorded throughout the 5-year follow-up, including decompensation events, hepatocellular carcinoma development, and survival. Crucially, HEV serology was performed only once at baseline, and data on repeat serology or seroconversion events during the follow-up period were not available. Patient inclusion was determined by the following strict criteria for CC:

Inclusion Criteria:

1. Age > 18 years at the time of HEV serology testing.
2. Confirmed diagnosis of liver cirrhosis (clinical, biochemical, and/or imaging/pathology).
3. CC diagnosis established through definitive exclusion of all known etiologies prior to June 2020.
4. Anti-HEV IgG serology test subsequently performed for any clinical reason.

Exclusion Criteria (Etiologies Required to be Ruled Out for CC Diagnosis):

CC was accepted as a diagnosis of exclusion only after the following conditions were definitively ruled out by liver biopsy, extensive laboratory testing, and detailed patient history:

- Viral Etiologies: Chronic Hepatitis B (HBV) (HBsAg negative), Chronic Hepatitis C (HCV) (Anti-HCV negative), and Hepatitis D (HDV).
- Autoimmune etiologies: Autoimmune hepatitis (AIH), primary biliary cholangitis (PBC), and primary sclerosing cholangitis (PSC) (negative/low-titer autoantibodies, including ANA, ASMA, LKM-1, and AMA; and exclusion by imaging/pathology).
- Alcohol-related liver disease (ALD): History of consumption exceeding >20 g/day for women or >30 g/day for men for >5 years.
- Metabolic/genetic etiologies: Evidence of hemochromatosis (HFE), Wilson’s disease (ATP7B), or alpha-1 antitrypsin deficiency (A1AT).
- Metabolic dysfunction-associated steatotic liver disease (MASLD/MASH): Definitive exclusion of MASLD/MASH based on imaging, elastography, or pathology showing absence of significant steatosis or MASLD-associated metabolic factors at the time of diagnosis.
- Other rare causes: Drug-induced liver injury (DILI) and vascular causes (e.g., Budd-Chiari Syndrome).

#### 2.1.2. Healthy Control Group

The healthy control group consisted of a historical cohort of 900 healthy blood donors whose data were previously reported by our institution [19]. These individuals were volunteers recruited from the blood bank at the same institution and confirmed to be healthy at the time of donation, with no history of chronic liver disease. The primary purpose of utilizing this large donor cohort was to establish the local HEV seroprevalence in the general healthy population for comparison against the CC patient group. Recognizing the potential for demographic differences (age and sex) between the historical control group and the CC cohort, these factors were planned to be addressed in the statistical analysis using multivariable logistic regression.

### 2.2. Data and Laboratory Analyses

Demographic data, existing anti-HEV IgG serology results, and the clinical and laboratory parameters of the patients were retrospectively extracted from electronic patient files and medical records. All included laboratory values for the CC patients were originally performed on venous blood samples collected after at least 8 h of fasting, as per standard hospital procedure. For the historical donor cohort, only the already existing an-ti-HEV IgG serology results were utilized.

The existence of HEV-specific IgG class antibodies (Ab) had been assessed using a solid-phase, indirect enzyme-linked immunosorbent assay (ELISA) kit (Euroimmun^®^, Lübeck, Germany) for all participants (both CC patients and the historical donor cohort). This particular assay employs recombinant HEV capsid proteins as the immobilized antigen to capture circulating IgG antibodies. The rigorous protocol provided by the manufacturer was strictly followed during the initial testing procedures, which included standardized incubation times, wash steps, and the use of the enzyme–substrate system for colorimetric detection.

Biochemical parameters, including aspartate aminotransferase (AST) (U/L), alanine aminotransferase (ALT) (U/L), total bilirubin (mg/dL), creatinine (mg/dL), albumin (g/dL), sodium (mEq/L), and international normalized ratio (INR), as well as hemogram data such as platelet count (×1000/mm^3^) were measured for the CC patients in the hospital laboratory using Roche Cobas C501 (Roche Diagnostics, Basel, Switzerland) and Beckman Coulter LH 780 (Beckman Coulter, Brea, CA, USA) analyzers. These existing results were subsequently retrieved and recorded for the purpose of this retrospective study.

Beyond the laboratory measurements, comprehensive clinical and historical data were meticulously retrieved from the patients’ medical records to fully characterize the CC cohort and support the diagnosis of exclusion. This included anthropometric data such as body mass index (BMI), detailed history of alcohol consumption (amount, duration, and pattern), presence of metabolic risk factors (e.g., diabetes mellitus, hypertension, dyslipidemia), and information related to potential HEV exposure risk factors, such as the history of consuming raw or undercooked pork products and wild game meat. Further-more, a thorough review of the medication history was performed to rule out any cases of drug-induced liver injury (DILI).

### 2.3. Liver Fibrosis Scoring

In patients with cryptogenic liver cirrhosis, FIB-4, MELD-Sodium, and APRI scores were calculated to assess the severity of liver fibrosis and cirrhosis. These scores were determined using the following formulas:

FIB-4 Score: Calculated using age, aspartate aminotransferase (AST), alanine aminotransferase (ALT), and platelet values.

MELD-Sodium Score: Calculated using bilirubin, creatinine, INR, and sodium levels.

APRI Score: Calculated using aspartate aminotransferase (AST) and platelet values.

### 2.4. Statistical Analysis and Ethical Approval

Statistical analyses were performed using SPSS (Statistical Package for the Social Sciences) version 21.0 (IBM, Chicago, IL, USA). Continuous variables were expressed as Mean ± Standard Deviation (Mean ± SD). The Chi-square (X^2^) test was used for comparing nominal variables between groups. Student’s *t*-test was applied for continuous variables with a non-normal distribution.

Regression Analyses

1. Determination of factors associated with Anti-HEV IgG seropositivity: Multi-variable logistic regression analysis was employed to independently assess the association between CC status and Anti-HEV IgG seropositivity, adjusting for confounding factors such as age and sex.
2. Evaluation of independent predictors for cirrhosis-related death: To evaluate the independent factors predicting the risk of cirrhosis-related death, a multivariable Cox regression analysis was performed, including Anti-HEV IgG seropositivity, age, sex, and the MELD-Na core.

In all analyses, a *p* value < 0.05 was considered statistically significant.

The study was approved by the Mersin University Clinical Research Ethics Committee, with the decision dated 27 August 2025, and numbered 2025/3223487.

## 3. Results

### 3.1. HEV Seroprevalence in Cryptogenic Cirrhosis Patients and Healthy Controls

The anti-HEV IgG seroprevalence was compared between the 52 patients with cryptogenic cirrhosis and the 900 healthy blood donors included in the study. Of the cirrhotics, fifty-three percent of the patients were female and 47% were male, with an average age of 65.21 ± 10.92 years. In the healthy blood donor group, the average age of the 900 volunteers was 35.22 ± 9.60 years, of whom 889 (98.7%) were male; the difference was statistically significant (*p* < 0.001).

**Figure 1:**
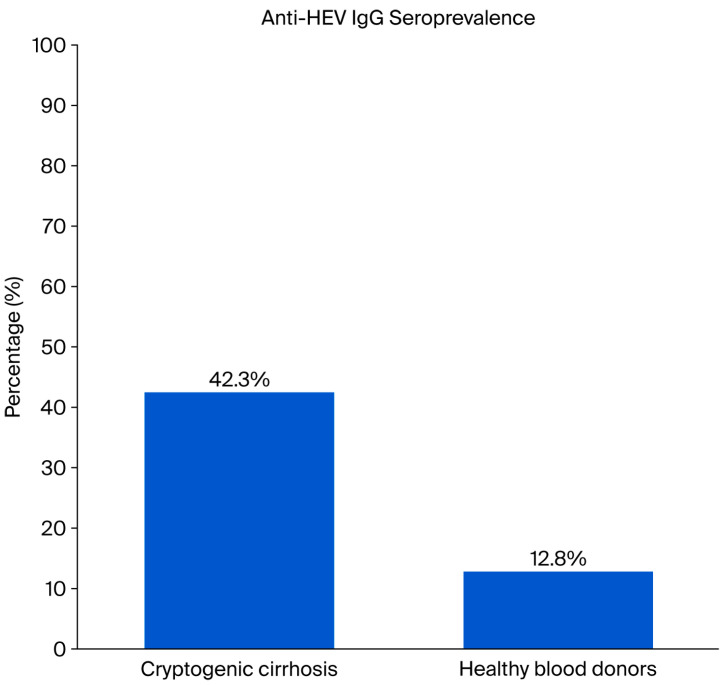
Comparison of Anti-HEV IgG Seroprevalence in Healthy Blood Donors (*n* = 900) and Cryptogenic Cirrhosis Patients (*n* = 52).

Anti-HEV IgG positivity was detected in 12.8% (*n* = 116) of healthy donors, whereas this rate was 42.3% (*n* = 22) in patients with cryptogenic cirrhosis. The seropositivity rate in patients with cryptogenic cirrhosis was statistically significantly higher than that in healthy blood donors (*p* < 0.001) (Figure 1).

The two groups were found to be statistically different with respect to age and sex (*p* < 0.001), necessitating adjustment for these potential confounders. Furthermore, other major known risk factors for HEV infection, such as a history of consuming raw or undercooked pork products were not present in the patient cohort. Therefore, a multivariable logistic regression analysis was conducted, adjusting for age and sex, to determine the independent effect of CC status on anti-HEV IgG seropositivity. The results confirmed that CC status (present/absent) remained an independent predictor of HEV seropositivity (Adjusted OR = 6.142, 95% CI: 2.221–16.984, *p* < 0.001), as detailed in Table 1). Numbered lists can be added as follows:

### 3.2. Comparison of Baseline Characteristics Between Anti-HEV IgG Seropositive and Seronegative Cryptogenic Cirrhosis Patients

Demographic, clinical, and laboratory data were compared between the Anti-HEV IgG-positive (*n* = 22) and negative (*n* = 30) groups of patients with cryptogenic cirrhosis. A statistically significant difference was found in age between the groups. The mean age of Anti-HEV IgG positive patients (69. 7 ± 7.2 years) was significantly higher than that of negative patients (61.7 ± 12.1 years) (*p* = 0.011) (Table 1).

No statistically significant difference was found between the two groups for bio-chemical parameters reflecting liver function, such as ALT, AST, total bilirubin, albumin, INR, sodium, and platelet counts (*p* > 0.05). Additionally, there was no statistically significant difference in the MELD-Na score, which indicates disease severity, between the groups (*p* = 0.086) (Table 2).

**Figure 2:**
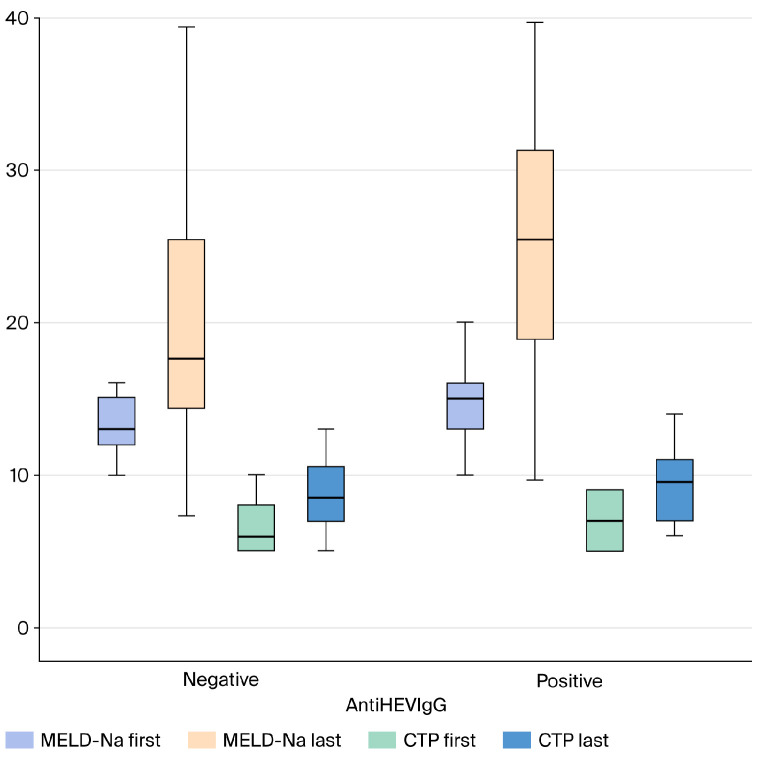
Comparison of Follow-up MELD-Na and CTP Scores in Cryptogenic Cirrhosis Patients Based on AntiHEV IgG Serological Status (Box Plot). Abbreviations: MELD-Na: Model for End-Stage Liver Disease–Sodium; CTP: Child–Turcotte–Pugh score; HEV: Hepatitis E Virus; IgG: Immunoglobulin G.

**Figure 3:**
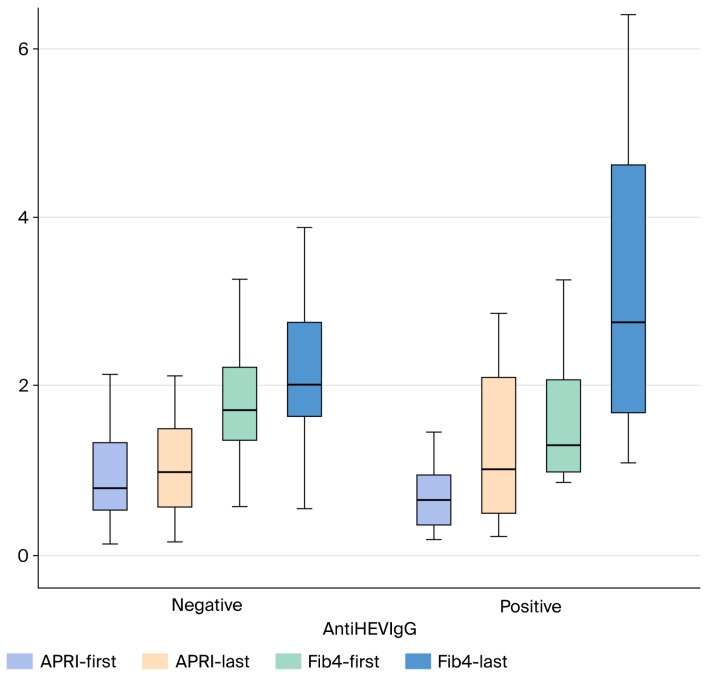
Comparison of follow-up APRI, and FIB-4 Scores in Cryptogenic Cirrhosis Patients Based on AntiHEV IgG Serological Status (Box Plot). Abbreviations: APRI: AST to Platelet Ratio Index; FIB-4: Fibrosis-4 score; HEV: Hepatitis E Virus; IgG: Immunoglobulin G.

APRI, FIB-4, and MELD-Sodium scores were compared between anti-HEV IgG-positive and -negative patients with cryptogenic cirrhosis. No statistically significant difference was found between the groups for the APRI (*p* > 0.05) and FIB-4 (*p* > 0.05) scores, two markers indicating the severity of liver fibrosis. Similarly, there was no significant difference in the MELD-Sodium score, which reflects disease severity and prognosis, between the two groups (*p* > 0.05) (Figure 2 and Figure 3).

### 3.3. Long-Term Data of Seropositive and Seronegative Cryptogenic Cirrhosis Patients

Cryptogenic cirrhosis patients (*n* = 52) were compared based on their anti-HEV IgG serological status regarding clinical endpoints and laboratory parameters during the five-year follow-up period (Table 3). During the five-year retrospective follow-up, overall mortality was observed in 14 of 52 patients (9 deaths in the anti-HEV positive group and 5 in the negative group). Of these, cirrhosis-related death was reported in 8 patients from the positive group and 4 patients from the negative group. Additionally, two patients—one from the positive group and one from the negative group—died due to non-cirrhosis-related causes (ischemic heart disease in both cases) (Table 3).

A statistically significant difference was found in the rate of death due to cirrhosis between anti-HEV IgG seropositive patients 36.4% and seronegative patients 13.4% (*p* = 0.047). The incidence of 4.6% vs. 0.0% and death due to other causes 4.6% vs. 3.3% did not show a statistically significant difference between the groups *p* = 0.423 and *p* = 0.820.

Regarding disease severity parameters, the MELD-Na score was statistically significantly higher in anti-HEV IgG seropositive patients mean 25.1 ± 7.74 compared to seronegative patients mean 19.6 ± 9.28 *p* = 0.029.

No statistically significant difference was observed between the groups in the mean values of other liver-function and fibrosis markers, including AST, ALT, Total Bilirubin, Albumin, INR, FIB-4 and, APRI scores (*p* > 0.05). Similarly, the CTP score *p* = 0.879 showed no significant difference between the groups.

A multivariable COX regression analysis was conducted to evaluate the independent effect of anti-HEV IgG seropositivity, age, gender, and MELD-Na score on cirrhosis-related death (Table 4).

The analysis showed that anti-HEV IgG seropositivity was not identified as a statistically significant independent risk factor for cirrhosis-related death after controlling for other variables (Hazard Ratio [HR]: 1.941, 95% CI: 0.563–6.695, *p* = 0.294).

Among the other variables included in the model, neither age (HR: 1.073, *p* = 0.055) nor the MELD-Na score (HR: 0.971, *p* = 0.400) significantly predicted the risk of cirrhosis-related death.

## 4. Discussion

Advances in our understanding of liver disease etiologies, the development of effective antiviral therapies against viral causes like Hepatitis B and C, and improvements in the early diagnosis and management of other liver diseases such as autoimmune hepatitis, primary biliary cholangitis, hemochromatosis, and Wilson’s disease have led to a relative increase in the proportional prevalence of cryptogenic cirrhosis (CC). For CC, whose etiology remains uncertain, a specific therapeutic approach beyond symptomatic treatment cannot be offered. Future research is anticipated to help clarify some of the underlying etiological factors of CC. In this context, the potential role of the Hepatitis E virus (HEV), which is now known to cause chronic infections in immunosuppressed individuals, in the etiology of CC is a subject of ongoing scientific inquiry. A notable gap in the current literature is the paucity of data on the precise etiological role of HEV in CC, and the virtually non-existent data regarding its long-term prognostic influence in this patient group. Therefore, our study addresses this knowledge gap by investigating HEV in CC patients in a twofold manner: assessing its etiological contribution (by comparing seroprevalence with healthy controls) and evaluating its long-term prognostic impact (by investigating its association with disease progression and major adverse clinical outcomes).

The most critical limitation of this retrospective study is its reliance solely on anti-HEV IgG serology to define exposure. As IgG positivity indicates only historical contact, it does not provide evidence of active or chronic viremia, which is hypothesized to be the true driver of accelerated liver disease progression. This absence of HEV RNA data represents a significant limitation that prevents us from determining the definitive causal role of active HEV infection in CC. Consequently, our prognostic conclusions must be interpreted within the context of past HEV exposure serving merely as a marker, rather than a definitive etiological agent.

### 4.1. Increased HEV Seroprevalence in Cryptogenic Cirrhosis

The primary finding of our study is the statistically significantly higher seroprevalence of anti-HEV IgG in patients with cryptogenic cirrhosis (CC) (42.3%) compared to healthy blood donors (12.8%) (*p* < 0.001). Crucially, the detailed analysis revealed that CC status (present/absent) remained an independent predictor of HEV seropositivity (Adjusted OR = 6.142, 95%: 2.221–16.984, *p* < 0.001), even after adjusting for the confounding effects of age and sex in the multivariable logistic regression analysis (Table 1). This result provides robust evidence for the potential, albeit complex, role of HEV exposure in the etiology or pathogenesis of CC.

This observation of a markedly high anti-HEV IgG seroprevalence in CC patients aligns with a substantial amount of literature exploring the association between HEV exposure and chronic liver disease. HEV infection typically causes a short, self-limiting disease, but its mortality rate can be as high as 70% in pregnant women and individuals with pre-existing chronic liver disease [20].

Other studies also support this potential link. For instance, a study from Sweden found HEV prevalence at 30% among 204 patients with hepatitis C infections and various stages of fibrosis, which was a statistically significant increase compared to the 17% anti-HEV IgG positivity rate found in blood donors [21]. Similarly, a case–control study con-ducted in Albania by Kondili et al. reported an anti-HEV prevalence of 36.6% in patients with chronic liver disease, significantly higher than the 12.1% in the control group [22]. This study also found that the presence of end-stage liver disease was independently associated with anti-HEV positivity [22]. Reinforcing the notion of an association between HEV exposure and severe liver pathology, an Argentinian study on patients with alcohol-related cirrhosis found that anti-HEV IgG seropositivity was seven times higher in the cirrhotic group compared to healthy controls [23].

However, not all studies have supported this relationship. A study that included a small cohort of patients found that although anti-HEV IgG positivity was numerically higher in the patient group (25.7% vs. 9.5%), the difference did not reach statistical significance [24]. These conflicting results underscore the variability in HEV epidemiology, a phenomenon heavily influenced by factors such as geography, the predominant HEV genotype, and public health standards. Our robust findings, which include adjustment for age and sex, therefore, emphasize the importance of regional studies and point to the need for a more nuanced understanding of this relationship.

### 4.2. Age, Immunosuppression, and Mechanisms of Chronicity

Our finding that anti-HEV IgG-positive patients with cryptogenic cirrhosis were significantly older than their negative counterparts (mean age: 69.7 ± 7.2 years vs. 61.7 ± 12.1 years, *p* = 0.011) (Table 2) is strongly supported by the literature, including studies by Faber et al., which showed that HEV seroprevalence increases with age [25,26]. This observation suggests a lifetime accumulation of risk of exposure to HEV or longer antibody persistence in the older population.

The literature clearly establishes that chronic HEV infection typically occurs in patients with severe immunosuppression, where a lower CD4-positive T-cell count, a lower HEV-specific T-cell response, and potent immunosuppressive treatments (e.g., tacrolimus use) have all been associated with chronic HEV infection [27,28,29]. Underlying chronic liver disease, including cryptogenic cirrhosis, can lead to a state of immune impairment often termed “cirrhosis-associated immune dysfunction” [24]. This compromised state, in turn, may create a vicious cycle, making individuals more susceptible to chronic HEV infection and potentially accelerating the progression of liver fibrosis and cirrhosis. The high seroprevalence we observed may thus reflect a cumulative effect of both past exposure and an inability to clear the virus due to disease-induced immune deficiency.

### 4.3. Comparison of Demographic Features and Liver Severity Scores by Anti-HEV Serology

Our study reveals that anti-HEV IgG seropositive cryptogenic cirrhosis patients were significantly older than their seronegative counterparts (mean age = 69.7 ± 7.2 vs. 61.7 ± 12.1 years, *p* = 0.011). This finding is highly consistent with the established general European and global literature, which consistently reports that HEV seroprevalence increases with age, reflecting cumulative lifetime exposure to the virus [18,26].

Importantly, however, we found no significant difference (*p* > 0.05) between the anti-HEV IgG positive and negative cryptogenic cirrhosis subgroups regarding classical liver function parameters such as ALT, AST, total bilirubin, albumin, and INR, or the fibrosis markers FIB-4 and APRI. Similarly, no statistically significant difference was observed in the baseline disease severity indicators, namely the MELD-Na score (*p* = 0.086) and the CTP score (*p* = 0.541). This outcome suggests that prior HEV exposure (IgG positivity), by itself, does not correlate with the current severity of liver disease or the stage of fibrosis.

This observation is supported by a study from Germany by Schulz et al., who investigated the seroprevalence of HEV in outpatients with CLD. They also reported a high prevalence of anti-HEV IgG antibodies but concluded that this positivity was not associated with the degree of liver fibrosis or the severity of the underlying liver disease as determined by FIB-4 and APRI scores [18]. Results similar to ours strengthen the argument that anti-HEV IgG positivity primarily reflects past immune response to the virus rather than an ongoing process of active liver injury.

Nevertheless, a crucial limitation of this interpretation is the absence of HEV RNA testing, which is a marker of active viremia. In contrast, retrospective studies, such as the one by Kamar et al. on organ transplant recipients, have found that chronic and active HEV infection was associated with rapid progression to cirrhosis [11]. Similarly, Barrague et al. also detected high HEV RNA levels in a patient with a non-alcoholic steatohepatitis background, with HEV RNA clearance achieved following Ribavirin treatment [30]. Our findings, which demonstrate a lack of correlation between anti-HEV IgG positivity and severity scores, imply that HEV’s contribution to chronic liver disease pathogenesis is complex and nuanced. These results support the hypothesis that HEV may contribute to the development of cirrhosis itself (as a cryptogenic factor), rather than acting as an independent predictor of its subsequent severity. Given the proven efficacy of Ribavirin treatment in chronic HEV infection [30,31], screening for HEV RNA in patients with cryptogenic cirrhosis and treating active infection, if found, could potentially halt or slow disease progression in this high-risk subgroup.

### 4.4. Impact of HEV Positivity on Disease Severity and Prognosis

This study is particularly relevant as it is one of the limited number of studies in the literature that specifically focuses on examining the long-term prognosis of cryptogenic cirrhosis patients in the context of anti-HEV IgG seropositivity. While HEV infection typically follows an acute, self-limiting course in immunocompetent individuals, HEV superinfection in patients with underlying chronic liver disease (CLD) has been robustly proven to accelerate prognosis deterioration, leading to decompensation and elevated mortality rates [32,33].

Our findings substantiate this general clinical observation with two key results in a cohort of patients with unknown etiology. First, we demonstrated that anti-HEV IgG seropositive cryptogenic cirrhosis patients had a statistically significantly higher baseline MELD-Na score (25.1 ± 7.74 vs. 19.6 ± 9.2, *p* = 0.029) compared to seronegative patients. The MELD-Na score is the strongest predictor of liver disease severity and short-term mortality risk. This discrepancy suggests that history of HEV contact is associated with a more severe and decompensated clinical phase in the natural history of these patients. This observation aligns with studies indicating that HEV seropositivity may play a role in the progression of cirrhosis particularly in HCV and HBV related cirrhosis [34,35]. Second, this severity difference manifested as a significantly higher rate of cirrhosis-related death (36.4% vs. 13.4%, *p* = 0.047) in the seropositive group. These univariable results suggest that HEV exposure could be an indicator of poor prognosis in cryptogenic cirrhosis patients.

However, the most crucial finding of our study clarifies the nature of this relation-ship: the independent effect of anti-HEV IgG positivity on cirrhosis-related death (HR: 1.941, *p* = 0.294) was lost in the multivariable COX regression analysis when controlling for strong confounders like MELD-Na and Age. This strongly suggests that the detrimental effect of HEV exposure on prognosis may be entirely accounted for by the patient’s pre-existing high disease severity (MELD-Na score), rather than a direct, independent chronic effect of the virus itself. This finding supports the consensus in the literature that the adverse prognostic impact of HEV superinfection is tightly linked to the degree of underlying liver disease decompensation [36]. Our results thus propose that anti-HEV IgG seropositivity serves as an indicator of a more advanced phase of cryptogenic cirrhosis, likely associated with pronounced immune dysfunction, but lacks independent prognostic power beyond the MELD-Na score [37].

### 4.5. Strengths and Limitations of the Study

While our study contains certain limitations, it also offers significant strengths that contribute to the current understanding of the complex relationship between HEV and CC.

First, we addressed a critical gap in the literature by simultaneously investigating both the etiological contribution (prevalence) and the long-term prognostic impact of HEV in this specific patient group.

Second, the five-year retrospective follow-up period provides valuable longitudinal data on disease progression (MELD-Na) and mortality, which is virtually non-existent for this specific patient cohort.

Third, the diagnosis of cryptogenic cirrhosis was strictly confirmed by applying exhaustive exclusion criteria for all other known etiologies of liver disease, thereby minimizing the potential for misclassification.

Finally, we employed robust multivariable statistical models, including Multivariable Logistic Regression to control for demographic confounders in the prevalence comparison and Multivariable Cox Regression to test for HEV’s independent prognostic effect on survival outcomes.

Despite these strengths, our study has certain limitations that warrant careful consideration.

The primary limitation stems from the retrospective, single-center design, which inherently limits the ability to establish causality and mandates replication in larger, prospective cohorts. Furthermore, our study is constrained by relying on a single baseline HEV serology test. The absence of repeated serological testing throughout the 5-year follow-up means we could not assess seroconversion events (HEV-negative to HEV-positive), which may have occurred and influenced long-term prognosis.

Most critically, the study relied solely on anti-HEV IgG serology to define HEV exposure. The absence of HEV RNA results in the available data prevents us from distinguishing between past exposure and acute or chronic active infection. Given that chronic viremia, rather than past exposure, is the hypothesized driver of accelerated liver disease in immunocompromised states, the precise mechanism of HEV’s prognostic contribution remains unresolved.

Furthermore, although adjusted for in the statistical analysis, the healthy control group displayed significant demographic differences in age and sex compared to the CC cohort, which may impact the generalizability of the prevalence comparison.

## 5. Conclusions

In conclusion, our study provides robust evidence that exposure to the HEV is an in-dependent factor strongly associated with CC. This is demonstrated by a significantly elevated anti-HEV IgG seroprevalence in CC patients, even after controlling for demographic confounders. Regarding prognosis, anti-HEV IgG positivity was linked to both a higher univariable mortality rate and a significantly accelerated progression of liver disease over the five-year follow-up, suggesting a detrimental effect. However, this association was not sustained as an independent risk factor for cirrhosis-related death in multivariable analysis. This suggests that HEV seropositivity may function as a marker for a more advanced stage of CC and underlying cirrhosis-associated immune dysfunction that predisposes both severe disease and HEV acquisition. To definitively clarify HEV’s true role in accelerating CC progression, and to determine if routine HEV screening (including HEV RNA) should be incorporated into the clinical management strategies for CC patients, rigorous prospective studies are essential.

## Figures and Tables

**Table 1:** Multivariable logistic regression analysis for factors associated with anti-HEV IgG seropositivity (adjusted for age and sex).

Variable (Predictor)	Odds Ratio (Exp(B))	95% Confidence Interval (CI)	*p*-Value
Age (years)	1.005	0.984–1.025	0.661
Gender (male/female)	0.595	0.223–1.589	0.300
Cirrhosis state (present/absent)	6.142	2.221–16.984	<0.001

**Table 2:** Baseline demographic, Clinical, and Laboratory Data in Patients with Cryptogenic Cirrhosis According to Anti-HEV IgG Serological Status.

	Anti-HEV IgG Negative (*n* = 30)	Anti-HEV IgG Positive (*n* = 22)	*p* Value
Age (years) (mean ± SD)	61.7 ± 12.1	69.7 ± 7.2	0.011
BMI (kg/m^2^)(mean ± SD)	24.9 ± 3.0	25.0 ± 2.4	0.911
AST (U/L) (mean ± SD)	45.4 ± 39.5	46.7 ± 27.5	0.894
ALT (U/L) (mean ± SD)	34.0 ± 35.5	31.1 ± 21.4	0.733
Total Bilirubin (mg/dL)	1.4 ± 0.9	1.3 ± 0.5	0.098
Sodium (mEq/L) (mean ± SD)	138.3 ± 3.5	135.6 ± 4.5	0.032
Albumin (g/dL) (mean ± SD)	3.6 ± 0.6	3.5 ± 0.6	0.409
Creatinine (mg/dL) (mean ± SD)	0.8 ± 0.2	1.0 ± 0.2	0.004
Hemoglobin (gr/dL) (mean ± SD)	11.8 ± 2.1	11.6 ± 1.9	0.803
WBC (/mm^3^)(mean ± SD)	5255.3 ± 2767.9	6398.8 ± 2266.2	0.169
Platelet (×1000/mm^3^)	124.5 ± 76.4	153.8 ± 64.4	0.175
INR(mean ± SD)	1.3 ± 0.4	1.4 ± 0.2	0.641
AFP ng/mL (mean ± SD)	3.1 ± 1.5	2.7 ± 2.0	0.551
CTP (A/B/C) (*n*/*n*/*n*)	18/11/1	10/12/0	0.341
CTP (mean ± SD)	6.71 ± 1.58	7.00 ± 1.66	0.541
MELD-Na (mean ± SD)	13.4 ± 2.33	14.8 ± 2.54	0.086
FIB-4 score (mean ± SD)	4.39 ± 2.71	5.02 ± 5.05	0.570
APRI score (mean ± SD)	1.11 ± 1.06	1.07 ± 1.11	0.894

Abbreviations: AFP, alpha-fetoprotein; ALT, alanine aminotransferase; Anti-HEV IgG, hepatitis E Virus immunoglobulin G antibodies; APRI, AST-to-platelet ratio index; AST, aspartate aminotransferase; BMI, body mass index; CTP, Child–Turcotte–Pugh score; FIB-4, fibrosis-4 score; INR, international normalized ratio; MELD-Na, model for end-stage liver disease–sodium; SD, standard deviation; WBC, white blood cell.

**Table 3:** Comparison of long-term clinical endpoints and prognostic parameters in cryptogenic cirrhosis patients according to anti-HEV IgG serological status.

	Anti-HEV IgG Negative (*n* = 30)	Anti-HEV IgG Positive (*n* = 22)	*p* Value
HCC *n* (%)	0/30 (0%)	1/22 (4.6%)	0.423
Death due to cirrhosis *n* (%)	4/30 (13.4%)	8/22 (36.4%)	0.047
Death due to other causes *n* (%)	1/30 (3.3%)	1/22 (4.6%)	0.820
AST (U/L) (mean ± SD)	47.7 ± 42.3	49.6 ± 30.8	0.884
ALT (U/L) (mean ± SD)	35.4 ± 37.4	25.8 ± 14.8	0.355
Total Bilirubin (mg/dL)	2.14 ± 1.89	2.65 ± 3.18	0.552
Sodium (mEq/L) (mean ± SD)	136.2 ± 7.6	137.8 ± 6.2	0.500
Albumin (g/dL) (mean ± SD)	2.87 ± 0.74	2.88 ± 0.70	0.981
Creatinine (mg/dL) (mean ± SD)	1.38 ± 1.51	1.24 ± 0.72	0.744
Hemoglobin (gr/dL) (mean ± SD)	10.2 ± 2.1	9.4 ± 4.9	0.243
WBC (/mm^3^)(mean ± SD)	6768 ± 5680	6256 ± 5043	0.169
Platelet (×1000/mm^3^)	124.1 ± 65.9	103.3 ± 64.0	0.175
INR(mean ± SD)	1.75 ± 0.86	1.77 ± 0.52	0.641
AFP ng/mL (mean ± SD)	4.2 ± 4.5	3.1 ± 2.4	0.551
CTP (A/B/C) (*n*/*n*/*n*)	6/12/12	4/7/11	0.763
CTP (mean ± SD)	8.63 ± 2.38	9.5 ± 2.44	0.879
MELD-Na (mean ± SD)	19.6 ± 9.28	25.1 ± 7.74	0.029
FIB-4 score (mean ± SD)	4.39 ± 2.71	5.02 ± 5.05	0.570
APRI score (mean ± SD)	1.51 ± 1.12	1.79 ± 2.37	0.579

Abbreviations: AFP, alpha-fetoprotein; ALT, alanine aminotransferase; Anti-HEV IgG, hepatitis E Virus immunoglobulin G antibodies; APRI, AST-to-platelet ratio index; AST, aspartate aminotransferase; BMI, body mass index; CTP, Child–Turcotte–Pugh score; FIB-4, fibrosis-4 score; HCC, hepatocellular carcinoma; INR, international normalized ratio; MELD-Na, model for end-stage liver disease–sodium; SD, standard deviation; WBC, white blood cell.

**Table 4:** Multivariable COX Regression Analysis for Factors Predicting Cirrhosis-Related Death in Cryptogenic Cirrhosis Patients.

Variable	Hazard Ratio	95% Confidence Interval (CI)	*p*-Value
Age (years)	1.073	0.563–6.695	0.055
Gender (male/female)	1.207	0.394–3.694	0.742
AntiHEV IgG (negative/positive)	1.941	0.563–6.695	0.294
MELD-Na	0.971	0.905–1.041	0.400

## Data Availability

The datasets generated and analyzed for this study are included in the published article. Further inquiries can be directed at the corresponding author.

## Abbreviations

The following abbreviations are used in this manuscript:

APRI	AST-to-Platelet ratio
HEV	Hepatitis E virus
FIB-4	Fibrosis-4 score
INR	International normalized ratio
MELD	Model for end-stage liver disease
